# Correction: A modified method to analyse cell proliferation using EdU labelling in large insect brains

**DOI:** 10.1371/journal.pone.0309825

**Published:** 2024-08-29

**Authors:** The PLOS ONE Staff

The first author’s initials appear incorrectly in the citation. The correct citation is Alcalde Anton A, Farnworth MS, Hebberecht L, Harrison CJ, Montgomery SH (2023) A modified method to analyse cell proliferation using EdU labelling in large insect brains. PLOS ONE 18(10): e0292009. https://doi.org/10.1371/journal.pone.0292009.

The publisher apologizes for the error.
