# Supplementary material for: A modified method to analyse cell proliferation using EdU labelling in large insect brains
Source: PLoS One. 2023 Oct 5;18(10):e0292009. doi: 10.1371/journal.pone.0292009 (PMC10553331; doi:10.1371/journal.pone.0292009)
Supplement: S1 File — (PDF) [file pone.0292009.s001.pdf]

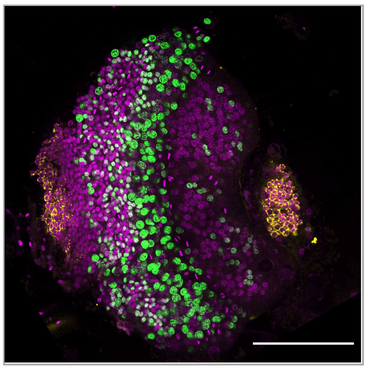

JUL 10, 2023

A modified method to analyse cell proliferation using EdU labelling in large insect brains

Amaia Alcalde Anton[1], Max S Farnworth[1], Laura Hebberecht[1], Jill Harrison[1], Stephen H Montgomery[1]

[1]School of Biological Sciences, University of Bristol, BS8 1TQ, UK

Amaia Alcalde Anton
University of Bristol

OPEN ACCESS

DOI:
dx.doi.org/10.17504/protocols.io.n92ldmy69l5b/v1

Protocol Citation: Amaia Alcalde Anton, Max S Farnworth, Laura Hebberecht, Jill Harrison, Stephen H Montgomery 2023. A modified method to analyse cell proliferation using EdU labelling in large insect brains. **protocols.io** https://dx.doi.org/10.17504/protocols.io.n92ldmy69l5b/v1

License: This is an open access protocol distributed under the terms of the Creative Commons Attribution License, which permits unrestricted use, distribution, and reproduction in any medium, provided the original author and source are credited

Protocol status: Working
We use this protocol and it's working

Created: Jul 04, 2023

Last Modified: Jul 10, 2023

PROTOCOL integer ID: 84483

DISCLAIMER

This protocol has been modified from the instructions of the Click-iT™ EdU Cell Proliferation Kit for Imaging, Alexa Fluor™ 488 dye - Thermofisher. Note that some steps (e.g. prepare the stock solutions) have not been changed.

ABSTRACT

The study of neurogenesis is critical to understanding of the evolution of nervous systems. Within invertebrates, this process has been extensively studied in *Drosophila melanogaster*, which is the predominant model thanks to the availability of advanced genetic tools. However, insect nervous systems are extremely diverse, and by studying a range of taxa we can gain additional information about how nervous systems and their development evolve. However, studying this variation requires adapting labelling techniques to visualise cell division in less commonly studied organisms, as methods developed for common laboratory insects often do not work. Here, we present a modified protocol for EdU staining to examine neurogenesis in large-brained insects, using Heliconiini butterflies as our primary case, but also demonstrating applicability to cockroaches, another large-brained insect.

IMAGE ATTRIBUTION

Amaia Alcalde Antón

SAFETY WARNINGS

Keywords: cell proliferation, neurogenesis, mitosis, neurodevelopment

EdU, 5-ethynyl-2'-deoxyuridine

- **Hazard**: Irritant
- **Exposure Route(s)**: inhalation, ingestion, injection, absorption
- **First Aid**: EYE CONTACT May cause eye irritation with susceptible persons. SKIN CONTACT May cause skin irritation in susceptible persons. INHALATION May be harmful by inhalation. INGESTION May be harmful if swallowed.

DMSO Dimethyl sulfoxide

- **Hazard**: Irritant
- **Exposure Route(s)**: Spills, splashing
- **First Aid**: SKIN CONTACT Rinse skin with water. Immediate medical attention is not required. EYE CONTACT Rinse cautiously with water for several minutes. Remove contact lenses, if present and easy to do. Continue rinsing.

Hoechst 33342

- **Hazard**: Mutagen
- **Exposure Route(s)**: Spills, splashing
- **First Aid**: SKIN CONTACT Rinse skin with water. Immediate medical attention is not required. EYE CONTACT Rinse cautiously with water for several minutes. Remove contact lenses, if present and easy to do. Continue rinsing.

DAPI - 4',6-Diamidino-2-phenylindole dihydrochloride

- **Hazard**: Irritant
- **Exposure Route(s)**: Spills, splashing
- **First Aid**: SKIN CONTACT Rinse skin with water. Immediate medical attention is not required. EYE CONTACT Rinse cautiously with water for several minutes. Remove contact lenses, if present and easy to do. Continue rinsing.

0. BEFORE YOU START – PREPARE THE STOCK SOLUTIONS

1 Prepare a 10 mM stock solution of EdU: Add 2 mL of DMSO to EdU, then mix well (store at ≤ 20 °C).

2 Prepare a working solution of the Alexa Fluor® azide: Add 70 µL of DMSO to the Alexa Fluor® azide (store at ≤ 20 °C).

3 Prepare a working solution of 1X Click-iT® EdU reaction buffer: Transfer all of the solution (4 mL) in the 1X Click-iT® EdU reaction buffer bottle to 36 mL of deionized water. Store at 4 °C.

4 To create the 10X stock solution of the Click-iT® EdU buffer additive, add 2 mL of deionized water to the vial (store at ≤ 20 °C).

5 Prepare paraformaldehyde, PFA (4% PFA in [0.1 M] phosphate-buffered saline (PBS; 7.4 pH). Alternatively, zinc-formaldehyde, ZnFA (0.25% [18.4 mM] ZnCl2; 0.788% [135 mM] NaCl; 1.2% [35

mM] sucrose; 1% formaldehyde) can also be prepared and used. Store at Room temperature
.

6 Prepare the permeabilization buffer (1 % Triton ® X-100 in [0.1 M] PBS). Store at 4 °C.

1. EDU INCORPORATION

7 Prepare the EdU working solution. Dilute EdU in Grace's Medium (GM, ThermoFisher, #11595030) to a final concentration of 20μM: add 20 µL from the stock solution (prepared in step 0.1) to 10 mL of GM. Make 1 mL aliquots (store at ≤ 20 °C).

8 A) ***Ex vivo*** brain incubation

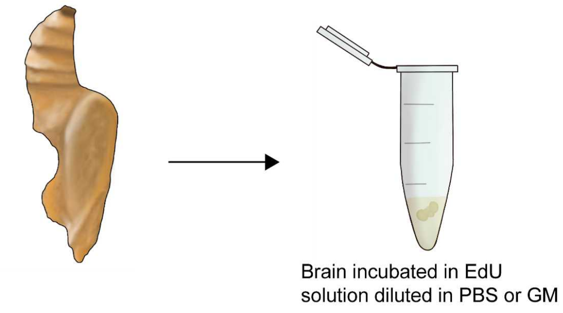

8.1 Dissect out the brain in 50% Grace's medium diluted in [0.1 M] phosphate-buffered saline (PBS; pH
7.4).

> **Note**
>
> This should be done as quickly as possible.

8.2 Incubate the brain in a solution of EdU diluted in Grace's medium for 1-2h.

Note

The time of incubation will affect the number of cell cycles which are exposed to EdU, and therefore longer incubations will likely lead to more labelled cells in mitotically active tissue.

9 B) ***In vivo*** incubation

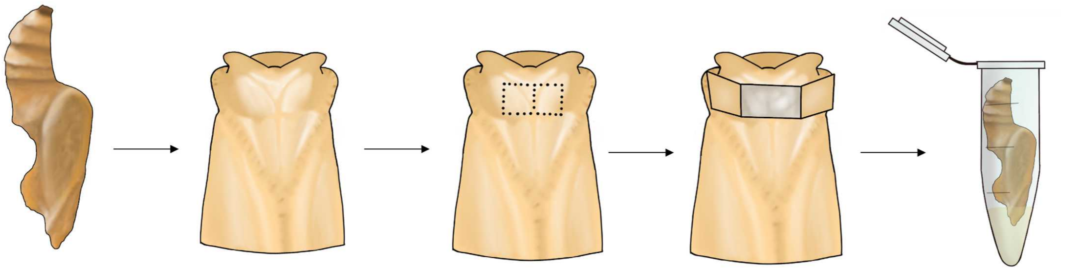

9.1 For active life stages, chill individuals in a refrigerator for a couple of minutes to anesthetize them. When ready, open a small window in the cuticle.

9.2 Incubate the brain in a solution of EdU diluted in Grace's medium for 1-2h.

9.3 After the incubation, finish the dissection in HEPES-buffered saline, HBS ([150 mM] NaCl; [5 mM] KCl; [5 mM] CaCl2; [25 mM] sucrose; [10 mM HEPES]; pH 7.4).

2. FIXATION

10 Transfer the brains to a 12 well plate, with up to 5 brains per well.

11 Fix the brain in a solution of paraformaldehyde, PFA (4% PFA in [0.1 M] phosphate-buffered saline (PBS; 7.4 pH) for 6-14h (Depending on the stage 14h for adults, 6-10h for larval stages).

Note

Alternatively, zinc-formaldehyde, ZnFA (0.25% [18.4 mM] $ZnCl_2$; 0.788% [135 mM] NaCl; 1.2% [35 mM] sucrose; 1% formaldehyde) can be used to fix the brains 14-16h (14h for larval brains, 16h for adult brains).

12 Remove the fixative and wash each well three times for 10 min with 1 mL of 0.1% Triton® X-100 in [0.1 M] PBS, 0.1%PBS-T.

Note

If tissue sectioning is required, we suggest performing it at this stage.

3. PERMEABILIZATION

13 Remove the PBS solution (from step 12).

14 Add 1 mL of 1% Triton® X-100 in [0.1 M] PBS, 1%PBS-T to each well.

15 Incubate at room temperature for 2h for whole brains, or 30 minutes for sectioned tissue.

4. CLICK IT REACTION

16 Dilute 1X Click-iT® EdU buffer additive in deionized water at a ratio of 1:10. This should be prepared **fresh** and used on the same day.

17 Remove the permeabilization buffer (from step 3.15).

18 Wash each well three times with 0.1% Triton® X-100 in [0.1 M] PBS, 0.1%PBS-T.

19 Remove the wash solution.

20 Prepare the Click-IT® reaction mix. Add the ingredients **in** the **order** defined by the manufacturer, replicated below. Use the Click-iT® reaction mix within 15 minutes of preparation.

Table 1. Click-iT® reaction cocktail. All the components should be added in the order listed below. Table from Click-iT® EdU Imaging Kits Protocol, ThermoFisher.

Reaction components	Number of wells (0.5mL/well)						
	1	2	4	5	10	25	50
1X Click-iT® EdU reaction buffer (from step 0.3)	430 µL	860 µL	1.8 mL	2.2 mL	4.3 mL	10.7 mL	21.4 mL
CuSO4	20 µL	40 µL	80 µL	100 µL	200 µL	500 µL	1 mL
Alexa Fluor® azide (from step 0.2)	1.2 µL	2.5 µL	5 µL	6 µL	12.5 µL	31 µL	62 µL
1X Click-iT® EdU buffer additive (from step 4.16)	50 µL	100 µL	200 µL	250 µL	500 µL	1.25 mL	2.5 mL
Total volume	500 µL	1 mL	2 mL	2.5 mL	5 mL	12.5 mL	25 mL

21 Add 0.5 mL of the Click-iT® reaction cocktail to each well containing up to 3 brains, and 1 mL to each well containing 4-5 brains. Make sure the brains are completely covered in the reaction mix.

22 Incubate the plate for 30 minutes at room temperature, **protect from light** using aluminium foil.

23 Remove the reaction mix.

24 Wash each well three times with 1 mL of 0.1% PBS-T (10 minutes each).

25 Remove the wash solution.

> **Note**
>
> Additional immunohistochemical staining can be performed at this point.

NUCLEAR STAINING with Hoechst 33342 or DAPI Staining

26 A) **Hoechst 33342**

26.1 Wash each well twice with 1 mL of [0.1 M] PBS (30 minutes each).

26.2 Remove the wash solution.

26.3 Dilute the Hoechst 33342 solution in [0.1 M] PBS at a ratio of 1:2000 to obtain a 1X Hoechst 33342 solution (the final concentration is 5 µg/mL).

26.4 Add 1 mL of 1X Hoechst 33342 solution per well. Incubate for 2-3 hours for whole brains, or 30 minutes for sectioned tissue, at room temperature, **protected from light**.

26.5 Wash each well twice with 1 mL of PBS.

26.6 Remove the wash solution.

27 B) **DAPI**

27.1 Wash each well twice with 1 mL of [0.1 M] PBS (30 minutes each).

27.2 Remove the wash solution

27.3 Wash once in milliQ/deionized H2O with 0.2% Triton, 10 minutes.

27.4 Dilute the DAPI solution in miliQ H2O at a ratio of 1:1000.

27.5 Add 1 mL of diluted DAPI solution per well. Incubate for 2-3 hours for whole brains, or 30 minutes for sectioned tissue, at room temperature, **protected from light**.

27.6 Remove the DAPI solution.

27.7 Wash each well with 1 mL of miliQ H2O with 0.2% Triton for 10 minutes.

27.8 Wash each well three times with 1 mL of 0.1% PBS-T.

6. TISSUE CLARIFICATION

2h

28

Note

Clarification methods can vary. In the current study we followed Ott (2008).

Incubate the brains under agitation in progressively more concentrated glycerol solutions: 1%, 2%, 4% (2 h each), 8%, 15%, 30%, 50%, 60%, 70%, and 80% (1 hour each) glycerol diluted in 0.1M Tris buffer, with DMSO to 1% final concentration.

29 Wash the brains with 100% ethanol for 30 minutes under agitation, repeat three times.

30 Transfer each brain in a small amount of ethanol (200-400 µL) to an Eppendorf tube.

31 Underlay the ethanol with methyl salicylate, wait for brain to sink with no agitation.

32 Aspirate the fluid from the top down, replace with fresh methyl salicylate and allow the brain to sink again for ~30 minutes with **no agitation**.

33 Aspirate the fluid and mount in fresh methyl salicylate.
