# Supplementary material for: A modified method to analyse cell proliferation using EdU labelling in large insect brains
Source: PLoS One. 2023 Oct 5;18(10):e0292009. doi: 10.1371/journal.pone.0292009 (PMC10553331; doi:10.1371/journal.pone.0292009)
Supplement: Attachment — Submitted filename: Response to Reviewers.docx [file pone.0292009.s003.docx]

**Additional Editor Comments:**

- *It should be explicitly pointed out what special modifications the new protocol has in comparison to the “standard EdU protocol”. The quality of Figures 4 to 6 should be improved (see comments from Reviewer #2). Furthermore, experiments to estimate the tissue penetration of the marker (buzzword: Z-stacks) as well as for autofluorescence in the nervous tissue of insects (buzzword: spectral scan) would be desirable. Finally, the reference list should be revised according to the suggestions of both Reviewers. Further suggestions for improvement can be found in the reports of the two experts (see below).*

Many thanks for this summary. We have responded to all these points below which we believe has improved the clarity and quality of the manuscript.

**Reviewer #1:**

- *The authors present a useful protocol for investigating neurogenesis during development of the insect nervous system using the EdU method. This method offers advantages over the main alternative, BrdU-labeling, in that denaturation of the DNA and immunolabeling are not required to identify proliferating cells. It is also likely that small fluorescent azide molecules penetrate tissues better than immunoglobulin molecules, making this method highly suitable for studying wholemount preparations of insect brains, although penetration was not directly assessed in this study and the photomicrographs included could all represent label in the superficial aspects of the tissue. If the authors collected Z-stacks and could estimate tissue penetration of the label, that would be very helpful. I appreciate the care the authors gave to identifying an effective method of delivering the marker to living tissue. My specific comments and questions follow.*

This is an interesting point. We note that in insect brains the majority of cell bodies are around the outside of the neuropils, so penetration is unlikely to be an issue for most cell types. Indeed, all scans were indeed from whole brains, so penetration has already occurred over reasonable distances. We have checked these images to identify cells within deeper tissue in the central brain. We now include a comment on this in the discussion (lines 249-253) and a table (S2 Table) showing the penetration of EdU staining at different stages of development.

- *It might be useful to non-specialists to include the common name of these butterflies (passion-vine or passion flower butterflies) and to note their Neotropical distribution.*

We have added this to lines 95, and 158-159. We have also added the common names of the other species mentioned: fruit fly, line (line 50), honey bee, (line 70) and red flour beetle (137).

- *2. I appreciate the scholarly overview provided by the citations, but was surprised to see Truman and Bate's 1988 (!) use of BrdU to study Drosophila neurogenesis not acknowledged (Truman JW, Bate M. Spatial and temporal patterns of neurogenesis in the central nervous system of Drosophila melanogaster. Dev Biol. 1988 Jan;125(1):145-57. doi: 10.1016/0012-1606(88)90067-x. PMID: 3119399.). Lovely to see the inclusion of Ruth Nordlander's and John Edwards' studies of monarch butterflies.*

Many thanks for pointing out this omission, we have added this reference to the line119.

- *3. To assist investigators just entering the field, define technical terms as fully as possible. Instead of "In comparison with other proliferation markers like pH3," make sure the reader knows the reference is to the use of anti-phospho-Histone H3 (Ser10) antibody as a marker for mitosis. Phosphorylation of Histone H3 begins during G2 but is reversed at the end of mitosis, so this label is a "snapshot," not a persisting marker. (Line 122)*

This is an important point, we have taken care to correct this and other instances.

- *4. Along the same lines, help the novice reading line 123 by replacing replication with S-phase.*

Done.

- *5. The test mentions both PFA and ZnFA, but the table, if I am reading it correctly, indicates that ZnFA never produced positive results. Can the authors clarify? The provided protocol is based on PFA but the authors mention that ZnFA can also be used. This is confusing.*

Thanks for spotting this, we have clarified this in the table. We have had positive results with ZnFA and PFA.

- *6. Did the authors perform a spectral scan to select markers that would minimize autofluorescence in insect nervous tissue?*

There is a degree of autofluorescence in our images, insect neuropils are known to autofluorescence around 480nm, and the Edu fluorescence was at 488nm. However, the autofluorescence does not appear in the cell nuclei, so this does not interfere with the use of Edu to label nuclei.

**Reviewer #2:**

- *1. This paper describes the adaptation of EdU protocol specifically for big insect species, EdU being very commonly used to label proliferating cells in many animal species including small insects. Although a very clear description of the protocol is given in supplementary materials, that will be useful for any researcher willing to use this technique, the manuscript suffers from weaknesses:*

We appreciate this positive comment. Indeed, we hope it will save other researchers working on large insects from unnecessarily investing time repeating the lengthy protocol optimisation that we had to perform. Indeed, we invested ~8 months trying alternative approaches before finalising the current approach.

- *2. The modifications performed compared to standard EdU protocol are not well underlined. It would help to point out in the text what was modified compared to standard EdU protocol to better emphasize the specificities of this new protocol*

Thank you for raising this, we have added a table (Table S3) introducing the main variations of this protocol and comparing the main steps with the manufacturer’s protocol and the protocol used in the *Drosophila* literature (e.g. Daul et al., 2010). The most important changes involve the incorporation of EdU, the fixation and permeabilization solutions, and the incubation times. The table is introduced in lines 245-246.

- *3. It would be interesting to illustrate the result of your protocol on Drosophila to compare the efficacy with standard protocol (identical results, better or worse?)*

We appreciate this point, but we are not aiming to present a more efficient protocol for all insects including *Drosophila*. Indeed, the *Drosophila* protocol seem to work well for that species and is more efficient in some regards, so it would seem unnecessary for *Drosophila* researchers to use our protocol. Several steps are also quite different because of the size of the tissue and the nature of the dissections, and this means a direct comparison with efficiency in *Drosophila* and large insects would be difficult to make. Our main goal is instead to present a working protocol for large insects, in other words in increase the breadth of efficacy rather than efficiency.

- *4. Fig4: labeling is blurred, does not look like confocal image (specially for Hoechst)*

Apologies, the images are somewhat pixelated due to insufficient resolution. We have replaced these images in a revised figure, and done the same for figure 3. We have also increased the magnification so the differences are clearer.

- *5. Fig 5 (cockroach) please add higher magnification picture*

Our intention was to show the overall cell cluster and the position of labelled cells within it. We therefore think the current figure meets this purpose. We have nevertheless also added higher resolution images of the nuclei as insets.

- *6. Fig 6 : the size of EdU labeled spots is too small to be nuclei. Does not look like in Fig3, where EdU clearly labels nuclei. Add Hoechst labeling to clarify*

Thank you, we have imaged other brains stained with Hoechst, EdU and HRP were the nuclei are marked more clear and revised the figure accordingly. We also include a higher resolution image.

- *7. Discussion: Any idea why EdU incorporation fails in big insect species while BrdU incorporation works (ex in Locusta migratoria, Periplaneta americana)? This first step (injection / incubation) is not different, only the revelation is.*

We note both do work, but with Edu the challenge has primarily been to increase uptake of the relevant chemicals. We are not sure why the success of the delivery differs between the methods. BrdU involves a lot of denaturisation steps which the Edu protocol does not and perhaps it is possible these steps help with the penetration of the secondary antibody. But if the reviewer has any suggestions to explain this difference we would be happy to discuss these in the manuscript.

- *8. Introduction : it would be fair to cite the first papers using BrdU to label neuroblasts in insect brain (Cayre et al Nature 1994 et Malaterre et al J Comp Neurol 2002)*

Indeed it would, apologies for that oversight. We have added these references on lines 119 and 120.

- *9. Error in references: ref 50 (Weng M, Komori H, Lee CY. 2013) cited page 7 does not mention the use of EdU*

Corrected, many thanks.
