# Supplementary material for: A modified method to analyse cell proliferation using EdU labelling in large insect brains
Source: PLoS One. 2023 Oct 5;18(10):e0292009. doi: 10.1371/journal.pone.0292009 (PMC10553331; doi:10.1371/journal.pone.0292009)
Supplement: Attachment — Submitted filename: Response to Reviewers.docx [file pone.0292009.s004.docx]

Review Comments to the Author

Please use the space provided to explain your answers to the questions above. You may also include additional comments for the author, including concerns about dual publication, research ethics, or publication ethics. (Please upload your review as an attachment if it exceeds 20,000 characters)

**Reviewer #1:**

- I enjoyed reading this manuscript a second time. I did not re-read with the intent to check every line, but I did note that there is a typographical error on line 289 (minutess instead of minutes).

Corrected, Thanks!

- Neuroanatomical studies on non-model organisms can be very challenging. I appreciate very much these investigators' careful work. I believe other research groups will find these methods useful.

Many thanks, that is our intention with this paper.

**Reviewer #2:**

- Thank you for providing better picture quality in this revised manuscript.

Thanks for the positive feedback.

- It is quite difficult for the reader to appreciate labeling efficiency since no explanations are provided concerning **labeled cells**: what are these labeled cells in which brain structure (mushroom bodies are mentioned in the introduction bot obviously cells labeled in Fig.6 are not Kenyon cells)?

Thank you for your response. Different figures show different brain regions. We have clarified this in the figure legends, lines: 279/fig 3 (the cells shown are in the developing optic lobes), line 295/fig 4 (shows the mushroom body) and lines 325-329/fig 6 shows a complete brain hemisphere of a 5^th^ instar larvae. The main point of this figure is to show that the protocol is compatible with immunohistochemistry.

To further elaborate, our goal is to validate the effectiveness of the protocol across large-brained insects, regardless of their brain structure. The cell identity is not a major concern as EdU marks every cell that undergoes the S-phase. This will be progenitor cells like neuroblasts, ganglion mother cells and intermediate proliferating cells; and their daughter cells, mainly neurons and glia. The efficiency should not be different in different cell types. The only potential limitation would be if the cell cycle duration exceeded the EdU incubation time, in which case some cell types might remain unmarked. In the present case we illustrate the protocol primarily by focusing on the mushroom body, while demonstrating that it works elsewhere in the brain, in different developmental stages and different insects. Other researchers could use it to mark cells anywhere in the brain of a large insect, or potentially other organs.

- Why so few cells in figs 4,5 and so many in fig. 6?

Thanks, that’s a good observation. This is because these figures are showing different developmental stages and different areas of the brain. We want to show that the protocol works in pupae but also in larvae. In figure 4, we are showing the mushroom body of a young butterfly pupa. We are here shortening the incubation, and this results in less cells marked. In contrast, in figure 6, we are showing a late larva (5^th^ instar). During this stage of development, a lot of proliferative cells are dividing and these are detected by EdU. It is a stage where many brain structures are changing/developing. Additionally, we show a full brain hemisphere with focus on the ventral part which still comprises several brain structures.

In figure 5, we are showing the mushroom body of a 4 week old cockroach, a different species and stage, so potentially fewer cells are proliferating, but this is not the intention of the comparison. We merely intend to show the protocol works in other large species.

- What does HRP label? Such information would help make sense of what is shown.

We indicated this in the methods section “As a primary antibody we used a rabbit antibody against horseradish peroxidase HRP, a common marker for neurons [52]. ”. We have also clarified this on the lines 323-324.
